# The Role of Molecular Microtubule Motors and the Microtubule Cytoskeleton in Stress Granule Dynamics

**DOI:** 10.1155/2011/939848

**Published:** 2011-06-20

**Authors:** Kristen M. Bartoli, Darryl L. Bishop, William S. Saunders

**Affiliations:** Department of Biological Sciences, University of Pittsburgh, 4249 5th Avenue, Crawford Hall 274, Pittsburgh, PA 15213, USA

## Abstract

Stress granules (SGs) are cytoplasmic foci that appear in cells exposed to stress-induced translational inhibition. SGs function as a triage center, where mRNAs are sorted for storage, degradation, and translation reinitiation. The underlying mechanisms of SGs dynamics are still being characterized, although many key players have been identified. The main components of SGs are stalled 48S preinitiation complexes. To date, many other proteins have also been found to localize in SGs and are hypothesized to function in SG dynamics. Most recently, the microtubule cytoskeleton and associated motor proteins have been demonstrated to function in SG dynamics. In this paper, we will discuss current literature examining the function of microtubules and the molecular microtubule motors in SG assembly, coalescence, movement, composition, organization, and disassembly.

## 1. Introduction

Stress granules (SGs) are nonmembranous cytoplasmic foci that rapidly appear in cells exposed to various types of stress including oxidative stress, heat shock, viral infection, and UV irradiation, all of which impair translation initiation (as reviewed in [1–3]). In response to such cellular insults, the cell activates mechanisms to selectively repress the translation of house-keeping gene transcripts to conserve energy for repair of stress-induced damage, while upregulating translation of proteins required for the repair process such as DNA-repair proteins, chaperone proteins, and transcription factors (as reviewed in [3–6]).

In this paper, we will discuss the importance of the microtubule network and the microtubule motors involved in SG dynamics including the assembly, coalescence, and disassembly processes. There are at least three different elements that are known to affect SG assembly and dynamics: posttranslational modifications, protein-protein interactions, and the microtubule network. The first two factors have been previously reviewed in [5, 7], and in this paper we will focus our attention on the role of the microtubule network and associated molecular motors in SG dynamics. 

Although the components of SGs often vary in different experimental systems or even under different types of stress, the formation of SGs is highly conserved [5, 7, 8]. SG formation has been observed in various systems including yeast [9–11], trypanosomatid [12], and mammalian [4, 13, 14]. Furthermore, SGs are not limited to in vitro cell culture models, as SGs have been observed in the tissues of stressed animals. Animals subjected to gentamicin-induced toxicity [15], ionizing radiation (IR) [16], and ischemic reperfusion injuries exhibit several hallmarks of SG formation [17, 18]. These results indicate that SG formation is part of a cell's native physiological response, rather than an in vitro artifact of cell culture systems.

At the onset of stress, SG formation typically begins through a mechanism involving phosphorylation of eIF2*α* mediated by one of four eIF2*α* kinases (PKR, PERK, HRI, or GCN2) [1, 5, 8, 14]. Each of these kinases are activated in response to specific types of stress, for example PKR is activated by double-strand RNAs in response to heat shock, UV, or viral infection [19, 20], PERK is activated in response to unfolded protein accumulation in the endoplasmic reticulum lumen [21, 22], HRI in response to oxidative stress [23], and GCN2 responds to amino acid deprivation and low nutrient levels [24]. eIF2*α* is a component of the ternary complex eIF2-GTP-tRNA_i_^MET^ required for translation initiation. Phosphorylation of eIF2*α* causes a reduction in the availability of eIF2-GTP-tRNA_i_^MET^, thus preventing assembly of the 48S preinitiation complex required for normal translation initiation [19]. The lack of available preinitiation complexes does not affect those transcripts actively undergoing elongation, allowing the translating ribosomes to terminate and run off, disassembling the polysomes [1, 2, 25]. The eIF2/eIF5 deficient “stalled” 48S preinitiation complexes consisting of bound polyadenylated mRNAs, the small ribosomal subunits, as well as various translation initiation factors including eIF3, eIF4E, and eIF4G, are organized into the newly forming SGs [26, 27]. These defective 48S preinitiation complexes are critical substrates for the assembly process of SGs to occur [26]. This formation is promoted through a variety of RNA-binding proteins (TIA-1/R [14], Fragile X Mental Retardation protein FMRP/FXR1 [28], G3BP [29], TTP [30], BRF1 [31], CPEB [32], and SMN [33]), some of which bind to RNA and oligomerize, leading to the initiation of SG assembly [3, 5]. 

Phosphorylation of eIF2*α* is not exclusively required for SG formation, as treatment with the translation initiation inhibitors pateamine A [34] or hippuristanol cause SG formation independent of eIF2*α* phosphorylation [35]. Both translation initiation inhibitors target the eIF4A helicase, thus preventing ribosome scanning and ultimately leading to inhibition of translation initiation and polysome disassembly [36, 37]. Polysomes and SGs are thought to be in equilibrium, as polysome stabilization with elongation inhibitors leads to disassembly of SGs even in the presence of eIF2*α* phosphorylation [5, 38]. Additionally, overexpression alone of specific RNA-binding proteins whose translation is typically repressed [5, 31, 32, 39, 40] or even the inhibition of certain initiation factors results in spontaneous SG formation [5, 41].

## 2. SG Dynamics

Within minutes of stress, the buildup of defective 48S pre-initiation complexes overwhelms the cells' ability to deal with the stalled translation, and cells begin to store these complexes temporarily [25]. This initiates the first stages of SG formation, where SGs begin to develop as small foci, which then coalesce or fuse to form larger foci that persist while the damage is repaired, and ultimately disassemble within a few hours of the stress recovery [4, 5]. As SGs disassemble, the mRNAs and associated proteins are able to resume normal translation.

The process of SG disassembly does not appear to simply be the process of assembly in reverse. Although certain markers of SG assembly/disassembly appear to correlate (i.e., phosphorylation status of eIF2*α*), the most compelling argument for distinct mechanisms of these two processes is that the series of events that occurs during the formation of SGs (small foci that coalesce into larger foci) is not reciprocated during the disassembly process. Rather, disassembly is observed as a dissolution of SGs rather than a breaking apart into smaller distinct foci [4, 5, 38]. These phenotypic differences are suggestive of different mechanisms underlying SG assembly and disassembly.  Table 1 lists proteins that associate or function with the cytoskeleton under nonstressed conditions and participate in SG dynamics. These proteins are characterized based on their function in SG dynamics as assembly factors, disassembly factors, or those proteins with additional functions in SG dynamics. 

Despite the fact that SGs can persist for several hours, SGs are highly dynamic. Fluorescence recovery after photobleaching (FRAP) experiments has demonstrated that certain components of SGs continuously exchange with cytosolic pools [7, 31, 38]. Some proteins stay associated with SGs until disassembly, however other proteins, exhibiting half-lives of 2–60 seconds, rapidly shuttle between the cytoplasm and SGs [4, 31, 38, 91–94]. Some of these rapidly shuttling proteins include RNA-binding proteins known to control mRNA stability, structure, and function (TIA1, TIAR-1, G3BP, PABP, HuR, and TTP) and those known to regulate mRNA translation and decay. This shuttling of proteins and mRNA in and out of SGs implies that they function as mRNA triage centers that sort sequestered mRNAs for storage, degradation, or translation reinitiation [5, 6, 95]. An RNAi screen completed by Ohn et al. identified over a hundred proteins which function in SG assembly [96]. Additionally, in a recent survey of the literature Buchan and Parker identified over 80 proteins which have been confirmed to localize to SGs [7]. Some of these proteins were not only found to be involved in mRNA metabolism, but some of which have functions in other cellular processes. Those proteins not involved in RNA metabolism span diverse functions as transcription factors, RNA-binding proteins, helicases, nucleases, molecular motors, and other signaling proteins [2, 3, 5, 7]. Because of this diversity, one cannot, with knowledge of protein function alone, predict if a protein will be found in SGs or not. This is exemplified by the fact that different experimental conditions produce SGs that contain different components.

## 3. SG Dynamics Require the Microtubule Network

Investigation of microtubule function in SG dynamics is made possible through the use of chemical inhibitors that allow disruption or stabilization of microtubules. Multiple investigations have concluded that the microtubule network is required for SG dynamics including the assembly, the coalescence, and the disassembly process as will be discussed below. Furthermore, recent data is emerging that is beginning to unravel which specific molecular microtubule motors participate in SG dynamics.

## 4. SG Assembly

Ivanov et al. were the first to investigate the effects of microtubule disruption on SG assembly [97]. Using the mammalian CV-1 cell line and two different inhibitors that depolymerize microtubules, nocodazole or vinblastine, a striking inhibition of SG formation in the presence of aresnite was observed. In agreement, Kwon et al. showed a similar phenomenon in HeLa cells following nocodazole treatment [43]. However, in contrast with these results, Kolobova et al., and Fujimura et al., amongst others, reported that microtubule disruption not only delayed SG formation, but when SGs did form, they were of significantly smaller size, greater in number and variable in distribution, rather than observing an abolishment of SG formation [44, 98, 99]. Some of these differences may be attributed to the concentration of the SG-inducing agent (arsenite) used, cell line-specific effects, or the markers used to measure SG formation.

SGs typically range in size from 0.2 to 5 *μ*m [1, 98, 100], however after microtubule disruption by nocodazole or colchicine, SGs were found to remain small (<0.6 *μ*m) [98], with a complete loss of large SGs [44, 98, 99]. Additionally, Kolobova et al. demonstrated that the number of individual SGs per cell was found to significantly increase, depending on the SG marker used [98]. Similar effects were observed by different groups in a variety of cell lines including CHO, HeLa, and COS-7 [44, 99, 100]. A decrease in SG size and an increase in SG number suggest a loss of SG coalescence in the absence of microtubules. These experiments demonstrate that microtubules are not required for the initial steps of SG formation, but are required for secondary steps in SG coalescence/aggregation [98].

Fujimura et al. confirmed the loss of SG coalescence after microtubule disruption by immunofluorescence while Kolobova et al. confirmed this coalescence defect by live cell imaging of cells transfected with mCherry-G3BP, a SG component, and RNA-binding protein [98, 99]. Depolymerization of microtubules led to decreased SG movement and an obvious loss in SG fusion/coalescence [98, 100]. Closer examination of fixed cells with various antibodies demonstrated changes in the composition of SGs [98]. A majority of SGs consistently contained many of the typical markers of SGs such as TIA1, G3BP, TIAR1, and other RNA-binding proteins [98, 99], but were deficient in a variety of proteins including CCAR-1, AKAP350A, eIF2*α*, HuR, and CUGBP, suggesting that loss of microtubules causes asymmetrical assembly of SGs, not just slowed assembly [8, 98, 99]. Therefore, coalescence is a microtubule-based phenomenon and is required to incorporate specific markers of SGs as the complete and proper assembly and maturation of SGs is affected after loss of microtubules. 

Typically, after SG formation and during the process of coalescence, the fully formed SGs are transported and organized around the perinuclear region [99, 100]. However after microtubule depolymerization, SGs were observed in fixed cells to be diffusely distributed throughout the cell in no discernable order, suggestive of microtubules functioning in transport and the reorganization process of SGs [44, 98–100].

**Figure 1:**
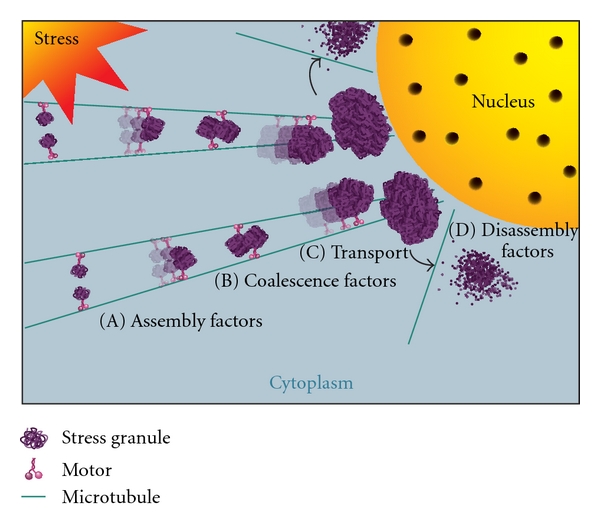
Model of microtubules and molecular motors in stress granule dynamics. When cells are exposed to stress-induced translational inhibition, SGs begin to form as small foci (A), assisted by assembly factors, and are transported along microtubules by molecular motors, enabling coalescence of SGs into larger foci (B). This process of coalescence continues while SGs are transported along microtubules to the perinuclear region where larger granules reside (C). Once the stress is removed SGs disassemble (D) aided by molecular motors as well as other disassembly factors. See Table 1 for the list of cytoskeletal proteins that function in these processes.

Investigation of SG transport by live cell imaging of CHO cells, selected for their distinct radial microtubule network, provided compelling evidence that SGs are indeed transported along microtubules [100]. Following double transfection of mCherry-tagged PABP and GFP-tagged tubulin revealed that SGs traveled along microtubules relocating to the perinuclear region [100]. This movement of SGs along microtubules tracked by time-lapse imaging was noncontinuous, but directional. SGs were found to move in one direction, stop, and sometimes travel again but not always in the same direction, consistent with the movement of microtubule motors. However, through its movement, the SGs continuously remained associated with microtubules, as confirmed in fixed cells [100]. When microtubules were disrupted, the dynamic movement of SGs was severely inhibited and ultimately SGs were not transported to the perinuclear region, confirming that microtubules are involved in the spatial intracellular placement of SGs. The above studies suggest that SGs are transported in a microtubule-dependent manner as small foci coalesce with other foci to form large SGs while on their way to their final destination of the perinuclear region (Figure 1). Loss of SG coalescence and variability in SG composition after microtubule depolymerization supports a microtubule-mediated transport of SGs.

SG assembly in the presence of the microtubule stabilizing drug paclitaxel was also investigated by three groups with different conclusions. Nadezhdina et al. showed an increase in SG movement when microtubules were stabilized and Ivanov et al. demonstrated that microtubule stabilization allowed for enhanced SG formation, favoring large SGs consistent with enhanced coalescence [97, 100]. However, Kwon et al. described microtubule stabilization as having no impact on SG assembly [43]. The reason for the discrepancy in these results is not immediately clear.

## 5. Disassembly

Microtubule function in SG disassembly has also been proposed [100]. SG disassembly has been shown to depend not only on the activity of HSP70 molecular chaperone that accumulates during stress induction, but also on the activity of certain phospho-specific proteins present in SGs [3, 8, 29, 52, 101]. Nadezhdina et al. took advantage of cycloheximides' ability to facilitate SG disassembly; cells treated with arsenite and cycloheximide disassembled SGs gradually starting 30 min after its addition. However, analysis of SG disassembly in cells treated with nocodazole prior to arsenite and cycloheximide treatment illustrated persistent SGs that did not disassemble or change in size [100]. This demonstrates a complete inhibition of SG disassembly when microtubules were disrupted and confirms the importance of the microtubule network in SG dynamics.

## 6. Molecular Microtubule Motors in SG Dynamics

Molecular microtubule motors are a class of proteins that transport intracellular cargoes along microtubules using ATP hydrolysis; there are approximately 50 different microtubule motors in humans. As discussed above, microtubules were shown to function in SG coalescence and disassembly processes; most likely through the association of microtubule molecular motors. Microtubule disruption resulted in a number of SG abnormalities including SG movement, coalescence, composition, relocation, and disassembly. Each of which can be explained through a molecular motor-based transport of SGs along microtubules towards the perinuclear region using SG coalescence to retain proper SG composition.

Recently, several publications have emerged demonstrating the importance of specific molecular motors in SG dynamics [43–45]. Loschi et al. identified the subunits of two molecular microtubule motors, cytoplasmic dynein and conventional kinesin, that localize to SGs and function in both SG assembly and disassembly [44].

## 7. Dynein

Dynein is a minus-end directed macromolecular motor complex functioning in numerous processes including mRNA movement [102], mitosis [103], and most recently SG assembly [44, 45]. Components of dyneins' intermediate chain (DIC) and heavy chain (DHC) were identified to localize to SGs by immunofluorescence analysis [44]. Upon closer examination of SG formation in the absence of dynein activity (by p50 overexpression [44], siRNA directed against DHC or DLC2 [44], or pharmacological-inhibition [43]) a ~3-fold impairment in SG formation was observed to occur after treatment with arsenite [43, 44]. The cells that contained SGs in the absence of dynein activity were found to be small in size and in number, suggesting that dynein is required for the initial stages of SG assembly [43, 44].

Studying SG dynamics in primary neurons by immunohistochemistry, Tsai et al. showed that dynein also functions in SG disassembly [45]. Additionally, assays measuring SG integrity by protease sensitivity of TIA-1 complexes [39] demonstrated that dynein enhances the integrity of SGs by assisting SG formation [45]. After transient expression of dynein, the TIA-1 complexes in stressed cells became more resistant to protease treatment than control-stressed cells, whereas in the absence of dynein, these TIA-1 complexes became more sensitive to proteases. These data demonstrate the importance of dynein function in SG dynamics in primary neurons not only in the assembly/disassembly process but also in the integrity of TIA-1 complexes [45].

## 8. Kinesin

Conventional kinesin subunits, KIF5B (kinesin heavy chain) and KLC1 (kinesin light chain 1), were also identified by Loschi et al. to localize to SGs and to function in SG dynamics [44]. Investigation of KIF5B in SG assembly after depletion revealed that this kinesin was not involved in SG formation; rather, after removal of arsenite treatment in cells depleted of KIF5B or KLC1, SGs were found to persist relative to control cells [44]. This suggests that conventional kinesin subunits, KIF5B and KLC1, participate in SG disassembly.

Motor proteins are known to work in concert, at times working together and at other times actively opposing each other to achieve the proper balance of transport. This coordinated relationship extends to SG dynamics as well. In the background of dynein knockdown, Loschi et al. was able to demonstrate that kinesin actually functions to inhibit SG formation. As mentioned above, the inhibition of dynein results in decreased SGs [43–45]. However, when cells were subjected to simultaneous inhibition of dynein (DHC1) and kinesin (KIF5B), SG formation resembled that of control cells [44]. Furthermore, the SG defects observed following KIF5B depletion were also partially reverted back to control cell levels following double knockdown, suggesting a possible redundancy of motors in the disassembly of SGs [44]. These results suggest that SG dynamics are in part orchestrated by a finely tuned balance of forces exerted by various molecular motors acting simultaneously.

## 9. Concluding Remarks

Not all of the defects in SG dynamics after loss of microtubules are mimicked by inhibition of either dynein or kinesin, individually or together. After impairment of dynein activity, a complete abolishment in SG formation was not observed, rather at least a third of the population of cells was still able to form SGs suggesting additional motors may participate in the assembly process [43–45]. Furthermore, neither depletion of dynein or kinesin was found to cause a loss in SG coalescence; this is in contrast to the disruption of microtubules which exhibited defects in SG coalescence. Although Tsai et al. speculated that the motor dynein plays a role in SG coalescence, the data is not as compelling or complete as its roles in assembly or disassembly [45]. Given that disruption of microtubules acts as a surrogate for pan-molecular motor inhibition, it is likely that other yet to be discovered molecular motors contribute to SG formation and/or participate in coalescence. Another motor, KIF18A, was identified in an RNAi-based screen to possibly function in SG assembly [96], but has not thoroughly been tested to date. Thus a continued search for molecular motors which participate in SG dynamics is needed to fully understand the underlying SG biology.

## Figures and Tables

**Table 1a:** (a) Assembly factors/phenotypes

Protein	Function	Cytoskeletal-association
CPEB	Translational regulator; OX induces SG formation; binding partners: RCK, eIF4E, FXR1P [32]	MT-associated [42]
DIC1/DHC1	Required for SG formation; transport [43–45]	MT-associated molecular motor [46]
DIS1	May function as a translational regulator; OX promotes assembly of SGs; binding partner eIF3h [47]	MT-associated [48]
eIF4A	An RNA helicase required for ribosome recruitment; inhibition induces SG formation [35]	Cytoskeletal-associated [49]
FMRP	Functions in mRNA transport or translation; OX induces SG formation; binding partners Ago2, RISC [28]	Cytoskeletal-associated [50, 51]
Grb7	Translational regulator; K/D inhibits SG formation; stimulates SG formation by stabilizing TIA-1 aggregates and enhancing SG or RNP integrity [52]	Cytoskeletal-associated [53]
Pumilio 2	Translational inhibitor; OX induces SGs; K/D interferes with SG formation [54]	MT-associated [54]
Smaug1	Translational repressor; OX induces SG formation [55]	Cortical cytoskeletal-associated [55]
Staufen	mRNA binding, transport, and decay; OX impairs SG formation; depletion facilitates SG assembly [56]	MT-associated [56, 57]
SMN	Assembly of small mRNP complexes; OX induces SG [33]	Role in actin dynamics [58]
TDP-43	OX induces SG; K/D has no effect on formation [59]	Role in microtubule organization [60]

**Table 1b:** (b) Disassembly factors/phenotypes

Protein	Function	Cytoskeletal-association
FAK	K/D impairs SG disassembly; FAK activity causes Grb7 phosphorylation and SG disassembly in recovering cells [52]	Role in actin assembly/microtubule organization [61, 62]
KHC/KLC	Required for SG dissolution; K/D inhibits SG disassembly; transport [44]	MT-associated molecular motor [63]
Dynein	Inhibition affects dissolution; transport [43–45]	MT-associated molecular motor [46]

**Table 1c:** (c) Factors with additional roles in SG dynamics

Protein	Function	Cytoskeletal-association
eIF2B	Guanine nucleotide exchange factor for eIF2 [27]	Cytokeletal-associated [64]
eIF3	Recruited to SGs during disassembly [5, 27]	MT-associated [49]
eIF4E	mRNA 5′ cap binding protein [65]	Cytoskeletal-associated [66]
eIF4G	Early translation initiation factor [67]	Roles in F-actin localization/microtubule organization [68]
FXR1P	FRMP-associated protein [28]	MT-associated [50]
FXR2P	FMRP-associated protein [28]	MT-associated [50]
Hsp27	Granzyme-associated molecular chaperone [69]	Cytoskeletal-associated [70, 71]
HuD	ELAV/Hu family of RNA-binding proteins, mRNA localization [72]	MT-associated [73]
NAT1/p97	Related to eIF4G, translational repressor activated in apoptosis [74]	MT-associated [75]
PABP-1	Initiation factor binds poly(A) tail [38]	Binds mRNA to MT [76]
PMR1	mRNA decay endonuclease forms a complex with TIA-1 [77]	Cytoskeletal-associated through binding partners [78]
RACK1	Allows survival of stressed cells [1, 79]	Required for astral MT length [80]
Sam68	Nuclear RNA-binding protein [81]	Roles in cytoskeleton organization [82]
TRAF2	Allows survival of stressed cells [1, 83]	Regulates actin cytoskeleton [84]
Xrn1	5′-3′ exoribonuclease I [31, 85]	MT-associated [86]
YB1	SG stability [87, 88]	Binds mRNA to microtubules [76]
ZBP1	RNA-binding protein important for localized translation [89]	MT-associated [90]
